# PARP1 is required for preserving telomeric integrity but is dispensable for A-NHEJ

**DOI:** 10.18632/oncotarget.26201

**Published:** 2018-10-05

**Authors:** Adam Harvey, Nicholas Mielke, Julia W. Grimstead, Rhiannon E. Jones, Thanh Nguyen, Matthew Mueller, Duncan M. Baird, Eric A. Hendrickson

**Affiliations:** ^1^ Department of Biochemistry, Molecular Biology, and Biophysics, University of Minnesota Medical School, Minneapolis, Minnesota 55455, USA; ^2^ Division of Cancer and Genetics, School of Medicine, Cardiff University, Heath Park, Cardiff CF14 4XN, United Kingdom

**Keywords:** PARP1, gene editing, NHEJ, HDR, telomeres

## Abstract

Poly-ADP ribose polymerase 1 (*PARP1*) is clinically important because of its synthetic lethality with breast cancer allele 1 and 2 mutations, which are causative for inherited breast and ovarian cancers. Biochemically, PARP1 is a single-stranded DNA break repair protein that is needed for preserving genomic integrity. In addition, PARP1 has been implicated in a veritable plethora of additional cellular pathways and thus its precise contribution(s) to human biology has remained obscure. To help address this deficiency, we utilized gene editing to construct genetically-null *PARP1* human cancer cells. We found a minor role for PARP1 in an alternative form of DNA double-strand break (DSB) repair, but only when these cells were deficient for the classical form of DSB repair. Despite being proficient for DSB repair, however, cell cycle progression defects and elevated endogenous DNA damage signaling were observed. These deficiencies were instead linked to telomere defects, where *PARP1*^−/−^ cells had short telomeres that co-localized with markers of endogenous DNA damage and were compromised in their ability to escape a telomere-driven crisis. Our data suggest that while *PARP1* does not participate significantly in DNA DSB repair itself, it does prevent the incidence of telomeric DSBs, which, in turn, can drive genomic instability.

## INTRODUCTION

Poly-ADP ribose polymerase 1 (PARP1) is a ubiquitously and very abundantly expressed protein that post-translationally modifies target proteins with poly-ADP-ribose (PAR) moieties using a nicotinamide adenine dinucleotide (NAD^+^) as its biochemical substrate. Impressively, the sheer abundance of these post-translational modifications in cells enabled researchers to discover such modifications prior to any information about the protein(s) responsible for their catalysis [1]. Of the 17 known PARP-domain-containing proteins (named PARP1 through PARP17, respectively), PARP1 is the most ubiquitous and most active isoform within eukaryotic cells, as its genetic deletion alone causes a dramatic loss in the amount of detectable PAR within cells [2]. Because PARP1 is the most abundant PARP and because PARylation is thought to be an important signaling process, it is perhaps not surprisingly that PARP1 has been implicated in a vast array of cellular processes, including cellular metabolism, cell cycle regulation, DNA replication, apoptosis and DNA break repair [3]. PARP1 has been comprehensively studied biochemically *in vitro* and extensively by genetic knockout *in vivo* in a plethora of model organisms including mice [4], plants [5], and flies [6], as well as in chicken DT-40 cells [7]. These reports generally conclude that PARP1 is important to preserve genomic integrity, and that it primarily participates in the repair of single-stranded DNA breaks (SSBs) [8]. To date there has been little phenotypic characterization of the genetic knockout of *PARP1* in human somatic cells, as the field has either relied on RNAi knockdowns, or, primarily, by utilizing one of the many inhibitors available to PARP1 [9, 10]. The use of RNAi, however, rarely completely eliminates the very abundant PARP1 from a given cell, potentially obscuring relevant phenotypes. In a complementary fashion, PARP inhibitors generally have a dominant-negative effect on cellular PARP1 by trapping PARP1 at a SSB in a DNA-bound state [11–13]. Thus, the normal role of PARP1 in human cells remains somewhat poorly defined.

*PARP1* is well-known because in its absence it exhibits synthetic lethality with breast cancer allele (*BRCA*)-deficient tumors [14, 15]. A prevailing theory (although other models have been proposed; see for example [16]) is that SSBs, which normally would be recognized for repair by PARP1, can accumulate over time and in a cancer cell such lesions would be converted to DSBs as a consequence of DNA replication [8]. Because *BRCA1* and *BRCA2* are required for the homology dependent repair (HDR) of these DSBs it has been postulated that it is this activity of *BRCA1-* and *BRCA2*-dependent repair that is required to preserve a viable level of genomic integrity in a PARP1-null/inhibited background [17]. This pathway, while clearly relevant to explain the impact of PARP1's absence on survival may, however, only be part of the story. For example, PARP1's association with the replication fork is required for checkpoint kinase 1 (Chk1)-dependent activation of checkpoints that enable repair of DNA damage encountered by the replisome during S-phase [18]. In addition to signaling that a replication problem exists, PARP1 is also required for the resolution of certain replication lesions. For example, when a replication fork encounters lesions or chromatin obstacles it can stall. One way to resolve the stalled fork is to reverse it into a so-called “chicken-foot” structure [19]. Fork reversal facilitates stabilization of the fork and likely provides a window of opportunity to initiate lesion/obstacle bypass [20, 21]. PARP1 is required for this replication fork reversal and in PARP1's absence, this reversal is blocked [22]. In another example, PARP1 has been shown to regulate replication fork speed and the inhibition of PARP1 can cause replication fork acceleration and subsequent related genomic instability [23]. Therefore, the absence of PARP1 might cause a higher frequency of lesions during S-phase in addition to dysregulating the DNA damage responses.

Besides impinging upon HDR, PARP1 has been additionally implicated in the repair of DSBs by actively regulating non-homologous end joining (NHEJ). NHEJ involves the end-to-end ligation of two broken ends of double-stranded DNA, and can be sub-categorized into Classic NHEJ (C-NHEJ) or Alternative NHEJ (A-NHEJ) pathways. The C-NHEJ pathway is absolutely dependent upon the Ku70/86 heterodimer, a ring-shaped protein complex that binds the ends of broken DNA. The binding of the ubiquitously expressed and very abundant Ku heterodimer and subsequent activation of the C-NHEJ pathway can occur within seconds of a DSB occurring and is inherently repressive of A-NHEJ [24–26]. Accordingly, A-NHEJ is thought to be a minor or back-up repair pathway in normal cells [27, 28]. The hallmark of A-NHEJ is the use of microhomology, which also constitutes a molecular signature that remains at the site of repair, to facilitate the ligation of the two DNA ends [29]. One documented pathological role for A-NHEJ is its likely involvement in oncogenic chromosomal translocations in mice [30], although this is probably not the case in human somatic cells [31]. PARP1 has been implicated in the regulation of A-NHEJ, which was partially a consequence of discovering that PARP1-associated proteins, such as X-Ray Cross Complementing 1 (XRCC1), were required for A-NHEJ [32–35]. Finally, it has been suggested that the DNA binding activity of PARP1 could compete with Ku to enable A-NHEJ to occur in place of C-NHEJ [32], or repress Ku's ability to access the DNA break [7]. In summary, PARP1 is clearly required for SSB repair and it appears to modulate DSB repair, although its role(s) in the latter pathway is still undefined.

One additional area of cellular biology where PARP1 may normally function is in telomere biology. Telomeres are the repetitive DNA:protein structures that serve to protect the ends of linear chromosomes from recognition as a DSB [36]. They are well known to regulate cellular aging, as they gradually shorten over time due to the end replication problem. Moreover, and of important clinical significance, the activation of a telomere re-elongation pathway is a key requirement for malignant progression [37]. Telomeres appear to be difficult regions of the genome to replicate [38], which is likely due to both the repetitive nature of the telomeric DNA, combined with their tendency to form G-quadruplex DNA [39, 40]. PARP1 has been identified as a telomere-binding protein and has been implicated in the regulation of telomere length maintenance [21, 41–44]. Confusingly, the influence of *PARP1* loss-of-function in the mouse has been reported to result in both telomere shortening [45] and to have no impact what-so-ever [46]. Similarly, there is a lack of agreement about the role of *PARP1* in telomere length maintenance studies in human cells as the use of either RNAi against *PARP1* [47] or inhibitors to PARP1 [47, 48] resulted in either telomere shortening [47] or lengthening [48]. Since some of these findings seem mutually exclusive and are likely due to differences in the experimental systems employed, a role for *PARP1* in mammalian telomere maintenance is still controversial.

To experimental address these issues, we utilized gene editing to generate *PARP1*-null human somatic cells. Human *PARP1*-null cells are viable but they exhibited spontaneous DNA damage, which tended to localize at telomeres, and was co-incident with short telomeres. Surprisingly, *PARP1*-null did not exhibit defects in DSB repair *per se*. Together, these data suggest that in human somatic cells PARP1's major role is in telomere maintenance and not DNA repair. This conclusion has significant relevance for clinical studies where the intervention of PARP1 activity is utilized.

## RESULTS

### Creation of *PARP1*-null cells

**Figure 1:**
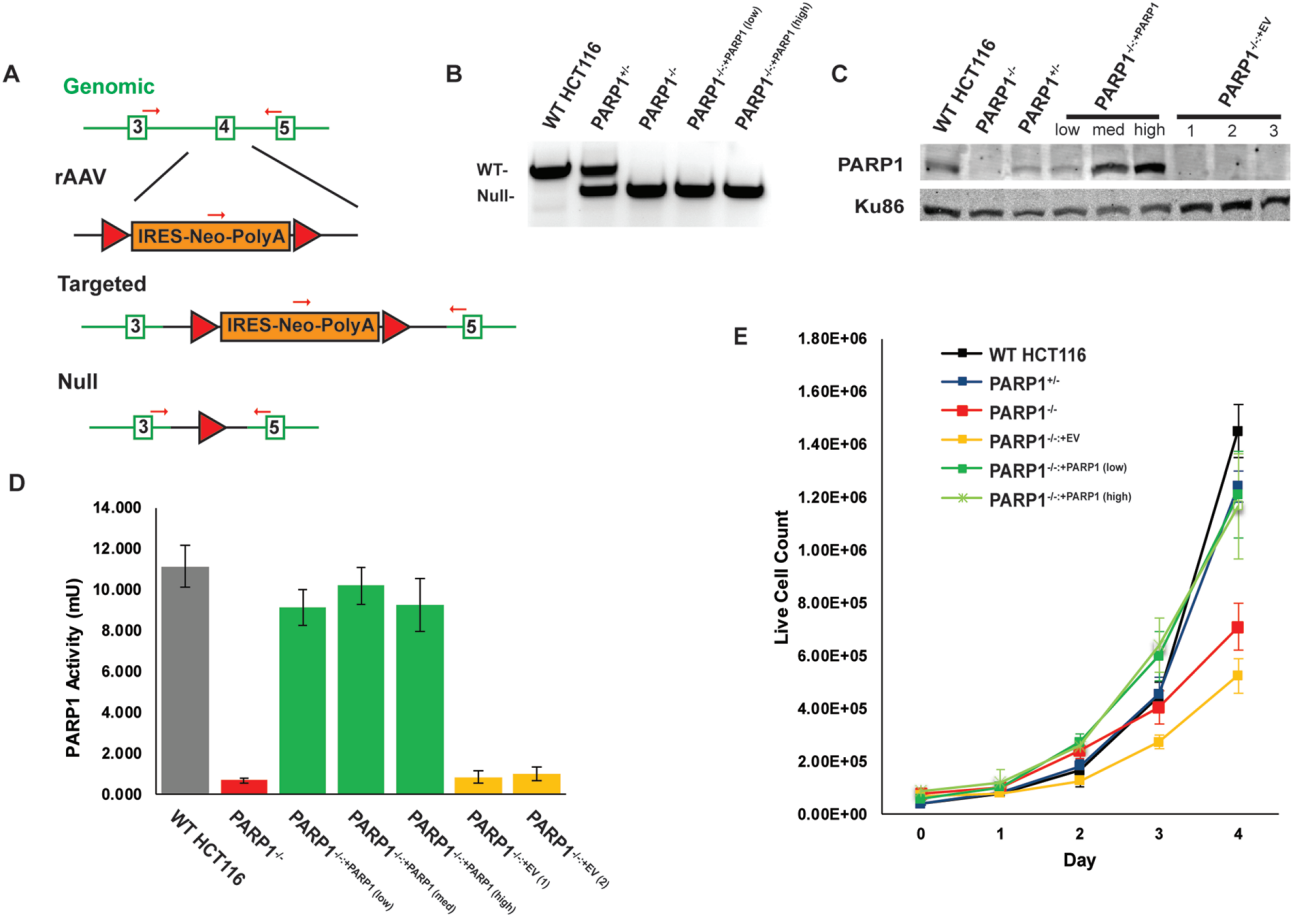
Construction and confirmation of *PARP1*-null cells **(A)** *PARP1* knockout HCT116 cells were constructed by rAAV-mediated gene targeting. Exon replacement of the 4^th^ exon (open green rectangle) of the *PARP1* gene with a floxed, Neo-cassette (orange rectangle) occurs by HDR, which can be subsequently removed by Cre-recombinase to result in the removal of the 4^th^ exon, causing a frame-shift mutation. Two rounds of gene targeting were performed to eliminate both alleles in this diploid cell line. Red arrows depict PCR primers used to monitor gene status. Red triangles represent LoxP sites. **(B)** PCR confirming the conversion of one wild-type (WT) allele to a null allele in a *PARP1*^+/−^ cell and the conversion of both wild-type alleles to null in *PARP1*^−/−^ null cells. **(C)** Western blot confirmation of the loss of PARP1 expression and confirmation of complementation of the null cells with PARP1 protein. **(D)** a PARP1 activity assay demonstrates that the wild type and indicated complemented cells exhibited WT-levels of parylation, while the null cells lacked such activity. EV is indicative of cells complemented with an empty vector. **(E)** growth curve depicting that the absence of PARP1 results in a slow growth phenotype.

We utilized gene targeting in human HCT116 cells to functionally inactivate *PARP1* by an exon-replacement strategy [49]. We designed a gene targeting construct in a recombinant adeno-associated viral vector (rAAV), such that correct targeting would result in the replacement of *PARP1* exon 4 with a *neomycin* drug selectable marker (Figure 1A). Correct gene targeting was screened for by using PCR primer pairs with one primer that flanked the targeting construct combined with an internal primer specific to the drug selectable marker. After successful gene targeting, the drug selectable marker was removed by Cre-recombinase, thus generating a null allele. Two rounds of such targeting were required to generate a diploid null cell line, which was confirmed using PCR primers that flank exon 4 (Figure 1B; Supplementary Figure 1A). In a scenario where a gene exhibits no strong selective pressure, Mendelian genetics would predict that when targeting a heterozygous cell line, there is an equivalent 50% chance of targeting either the already targeted allele (“re-targeting”) or targeting the second, still functional allele. During the second round of *PARP1* targeting, only 3 of 72 correctly targeted clones resulted in the loss of the second *PARP1* allele (*i.e*., 69 of 72 clones were re-targeted) (Table 1). This exceptional disequilibrium in the gene targeting frequency is usually a hallmark of genes that provide a significant growth disadvantage when absent [50–52]. Thus, although the isolation of three independent *PARP1*-null clones was unequivocal evidence that *PARP1* is not essential in human HCT116 cells, the frequency with which these clones were obtained was also an indication that *PARP1* has an important role in human cellular biology.

**Table 1:** PARP1 gene targeting results

Desired Genotype	Targeted/Random Insertion	Number of Targeted Clones	Expected Number of Desired Clones
Parp1^+/−^	23/96	23	Not Applicable
Parp1^−/−^	72/139	3	36

We next sought to complement these cells with either an empty vector, or a wild type (WT) *PARP1* cDNA, which was integrated randomly into the genome by a PiggyBac transposon system [53]. The restoration of PARP1 protein in the null cells was confirmed by a western blot analysis (Figure 1C; Supplementary Figure 1C). A series of complemented clones in which PARP1 was either under- (“low”), ~normally- (“medium”) or over- (“high”) expressed were generated for each of the three null cell lines (Figure 1C; Supplementary Figure 1C). In order to validate that the complemented clones contained active protein, a PARylation assay was performed, which confirmed both the successful ablation of PARP activity in the null cells, as well as their functional complementation (Figure 1D). Interestingly, even the lowest levels of complementing PARP1 expression could fully rescue PARP activity (Figure 1D). An obvious phenotype of the *PARP1*^−/−^ cells was their slow growth, as they exhibited an almost 50% reduction in doubling time (Figure 1E; Supplementary Figure 1B). Again, this phenotype could be completely rescued by the re-expression of even low levels of PARP1 (Figure 1E). In summary, these data compellingly demonstrated that *PARP1* is not essential in human somatic cells, but that its absence results in significant deficits to both replication and survival.

### *PARP1*-null cells accumulate in G_2_ of the cell cycle

**Figure 2:**
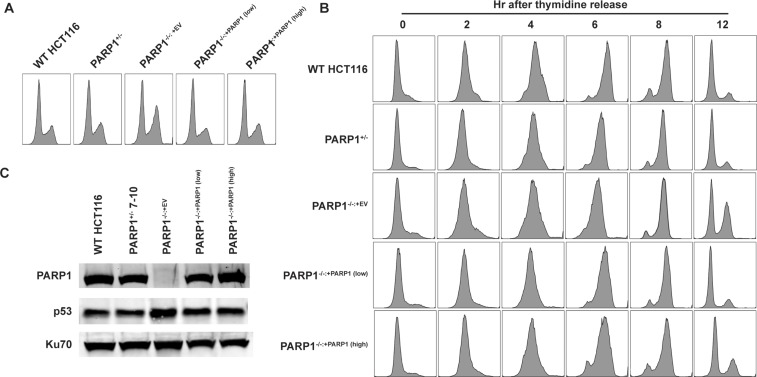
*PARP1*-null cells exhibit a G2-growth arrest **(A)** the DNA content of asynchronously growing cells exhibited a modest, constitutive G_2_ cell cycle accumulation. **(B)** a time course study of thymidine-block synchronized cells. After release, all cells appeared to progress through S-phase at approximately the same rate, but many *PARP1*-null cells did not progress through mitosis, but rather exhibited a G_2_/M cell cycle accumulation. **(C)** Western blot evidence for increased p53 expression in PARP1^−/−^ cells. Ku70 was used as a loading control (Supplementary Table 2).

In order to better understand the cellular growth defect, we investigated whether this was correlated with deficits in cell cycle progression. In asynchronous populations, the null cells exhibited a modest increase in the number of cells in G_2_, compared to both WT and the complemented cells when the DNA content of these cells was analyzed using propidium iodide staining (Figure 2A). In order to better understand this G_2_ accumulation, cells were synchronized at the G_1_/S transition point with serum starvation, followed by an overnight incubation in thymidine (in the presence of serum), which transiently arrested the cells at the G_1_/S transition. After releasing the cells into standard media, the cell cycle profile of the cells was determined (Figure 2B). The *PARP1*-null cells progressed through the cell cycle at approximately the same rate as either WT or complemented cells, but after S-phase, dramatically accumulated in the G_2_ phase. This is best exemplified by the amount of *PARP1*-null cells remaining in G_2_ (36.4%) at the 12 hr time point, in comparison to the rest of the genotypes (12% to 18%), which were successfully able to continue through mitosis and into the subsequent G_1_ phase (Figure 2B). The accumulation of *PARP1*-null cells in the G_2_-phase of the cell cycle, suggested that the cells were experiencing an elevated level of DNA damage that was, in turn, activating the G_2_/M checkpoint. Consistent with this interpretation, *PARP1*-null cells had elevated levels of p53, with respect to control lines (Figure 2C; Supplementary Figure 2).

### *PARP1* modulates, but is not required for, A-NHEJ

**Figure 3:**
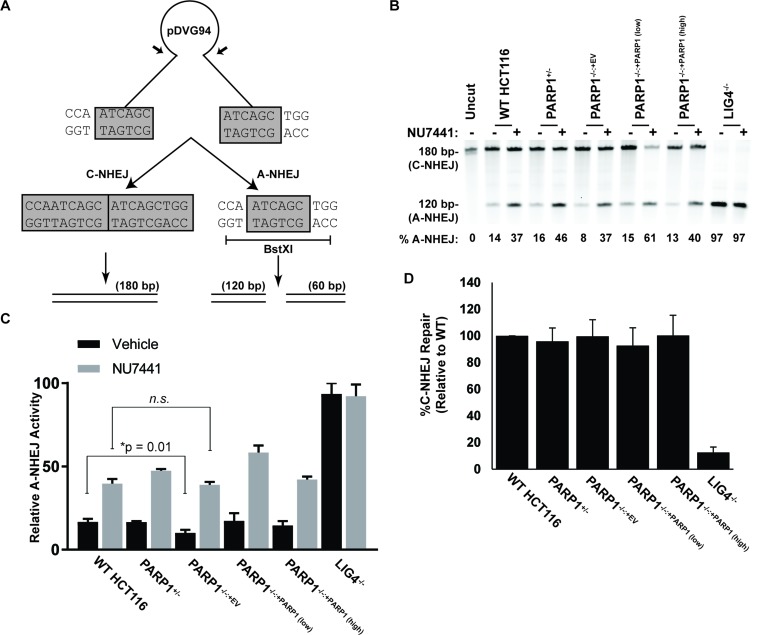
The impact of the absence of PARP1 on A- or C-NHEJ **(A)** schematic of the pDVG94 plasmid. **(B)** the indicated cell lines were treated with 1 μM of the DNA PK_cs_ inhibitor, NU7441, for 4 hr, and then transfected with linearized pDVG94. Cells were allowed 24 hr to repair the linearized template (still in the presence or absence of inhibitor), then plasmids were extracted, and the region spanning the cut site was amplified by PCR, followed by digestion with *BstXI*. The restriction enzyme products were then analyzed by agarose gel electrophoresis. The gel fragments corresponding to either C-NHEJ- or A-NHEJ-mediated repair migrate either at 180 or 120 bp, respectively. **(C)** quantitation of three experiments similar to panel B. *PARP1*^−/−^ cells exhibited a significant (p = 0.01) ~2-fold reduction in baseline A-NHEJ activity but were not statistically different from wild type cells under induced conditions. **(D)** the indicated cell lines were transfected with linearized pGEM-Ad2-EGFP plasmid and subjected to flow cytometry analysis. Only DNA *LIG4*^−/−^ cells exhibited a significant defect in DNA repair.

The spontaneous elevation of p53 expression in asynchronously growing *PARP1*-null cells suggested that the absence of *PARP1* was contributing to an accumulation of DNA damage. Given the aforementioned reports of PARP1's role in DSB repair, we thus next measured the cells' capacity for this activity. Cells were transfected with a linearized plasmid-reporter, pDVG94, allowed 48 hr to enact repair, and then circularized plasmids were recovered from the transfected cells. Cells have two options to repair the linearized plasmid. They can simply re-ligate the ends together, which is indicative of C-NHEJ and which can be quantitated as a ~180 bp PCR product when primers flanking the repair junction are utilized (Figure 3A). Alternatively, cells that utilize the 6 bp of microhomology present at the linearized ends to repair the plasmid create a diagnostic restriction enzyme recognition site, *BstxI*. Cleavage of the PCR products generated with primers flanking the repair junction with *BstXI* generates a 120 bp fragment (and a 60 bp fragment) and the appearance of this product(s) versus the 180 bp product enables a relative measure of A-NHEJ versus C-NHEJ activity (Figure 3A) [54]. In wild type cells approximately 15% of the repair products could be ascribed to A-NHEJ (Figure 3B, 3C). As a positive control we also analyzed the same plasmid rejoining in a cell line defective in DNA Ligase 4 (*LIG4*) [55]. As expected [24, 25] these cells carried out virtually exclusively (97%) A-NHEJ (Figure 3B, 3C; Supplementary Figure 3). Consistent with previous studies [32–35], the *PARP1*-null cells showed a statistically significant deficit in A-NHEJ (Figure 3B, 3C). The deficit, however, was rather small (<2-fold), and importantly could not be phenocopied by treating wild type cells with a PARP1 inhibitor, olaparib (Supplementary Figure 3). To clarify these results, we next induced A-NHEJ activity by pretreating cells with the DNA-PK_cs_ inhibitor, NU7441 [56], which should increase the relative amount of A-NHEJ by inhibiting the Ku/DNA PK_cs_-dependent C-NHEJ pathway. All genotypes, except again as anticipated, *LIG4*-null cells, showed enhanced A-NHEJ activity in the presence of NU7441 (Figure 3B, 3C). Importantly, however, the *PARP1*-null cells showed increases in A-NHEJ activity comparable to the wild-type and complemented clones (Figure 3B, 3C). Most provocatively, the treatment of *LIG4*- and *DNA-PK_cs_*-null clones with olaparib was completely ineffective in inhibiting A-NHEJ (Supplementary Figure 3). Thus, the absence of PARP-1 did not affect the cellular capacity for A-NHEJ in all situations where A-NHEJ activity was either genetically or chemically enhanced.

Since previous models had suggested that PARP1 may compete for DSBs with the Ku heterodimer [7, 32] we carried out an additional experiment to test whether or not the converse of our conclusion that PARP1 activity might be C-NHEJ dependent was true: *i.e*., whether the absence of PARP1 affects C-NHEJ. *PARP1*^−/−^ cells and relevant controls were transfected with the pEGFP-Pem1-Ad2 reporter, which measures C-NHEJ activity [57]. As expected, a LIG4-null cell line showed greatly reduced activity in this assay (Figure 3D; Supplementary Figure 4). In contrast, the presence or absence of PARP1 had no effect on the levels of C-NHEJ in the various cell lines (Figure 3D; Supplementary Figure 4). Thus, we conclude that PARP1 does not participate (significantly) in either C-NHEJ- or A-NHEJ-mediated DSB repair in human cells.

### PARP1 is required for proper telomere maintenance

**Figure 4:**
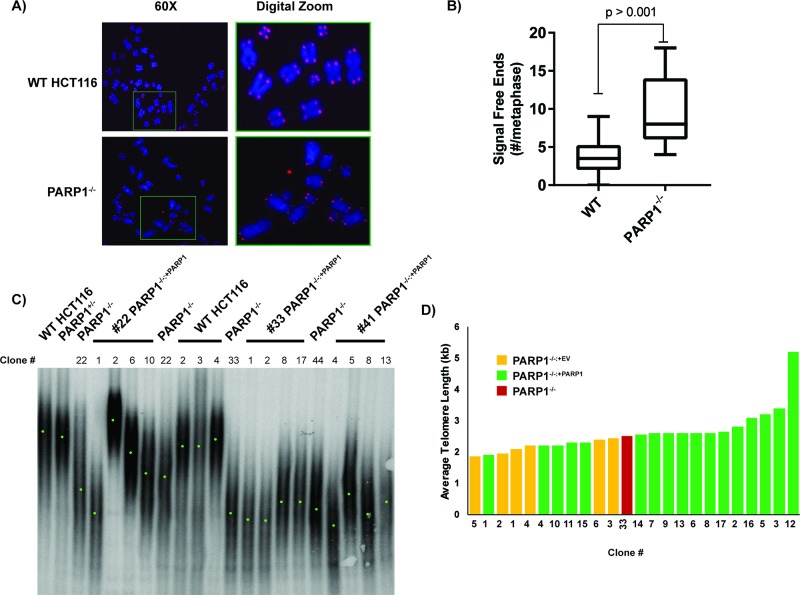
*PARP1*-null cells exhibit telomere dysfunction **(A)** *PARP1*-null cells have an increased frequency of signal free ends. Metaphase spreads were prepared from the indicated cell lines and then stain with a telomeric PNA probe (red spots) and then counter-stained with DAPI (blue). **(B)** the number of signal free ends from ~25 such metaphases was quantified for each genotype. **(C)** a TRF analysis of the telomere length of the indicated cell lines. For many of the cell lines, independent subclones were isolated and these are indicated by the clone number. Genomic DNA from the indicated cell lines was prepared, digested to completion with frequent cutting restriction enzymes and the residual DNA was electrophoresed onto an agarose gel and then transferred to nitrocellulose. The blot was subsequently hybridized with a radioactive telomeric probe. Since telomere length is variable from chromosome end to chromosome end and from cell to cell, a smear results. The mid-point of the telomeric smear is indicated with a green point. **(D)** *PARP1*^−/−^ cells have short telomeres, which can be rescued by complementation. In order to evaluate the clonal effect of telomere length, we derived empty vector containing (yellow rectangles), and complemented clones (green rectangles), from a given parental null clone (red rectangle) and then determined their telomere length by TRF analysis as shown in (C). The average telomere length by as determined by densitometry is shown.

The above experiments demonstrated that while *PARP1*-null cells seemed to be sensing significant amounts of DNA damage or stress, they had only mild deficits in the repair of such damage. Thus, we hypothesized that this damage might rather be associated with stalled or stressed DNA replication forks [23]. One region of particular interest was telomeres [58], as PARP1 had been identified as a telomere-binding protein, and some previous reports of telomere shortening have been associated with PARP1 inhibition or inactivation [41–44]. To explore this possibility, we — in a blinded fashion — analyzed 50 randomly-selected metaphase spreads from wild type and *PARP1*^−/−^ cells for the presence of signal-free ends. After un-blinding the images, 26 were wild type, and 23 were from *PARP1*^−/−^ cells (one image was discarded due to poor quality). Signal-free ends are operationally defined as telomeres that are so short that they do not hybridize well to a telomere-specific [(A_2_TC_3_)_3_] fluorescent protein:nucleic acid (PNA)-probe (Figure 4A, 4B). Cells with normal length telomeres will generally yield a uniform staining pattern with 4 red fluorescent spots — one at the ends of each chromatid (Figure 4A). In contrast, *PARP1*^−/−^ metaphases exhibited variable staining and a significant increase in the number of chromatid ends where no hybridization signal was visible whatsoever (Figure 4A, 4B). Thus, *PARP1*^−/−^ cells appeared to have at least a subset of very short telomeres. Deficits in telomere length were confirmed by Southern blotting. Initial screening of several subclones of WT, *PARP1*^−/−^, and *PARP1*^−/−:+PARP1^ complemented cells demonstrated that the null cells (median telomere length of 2.5, 2.1, and 2.3 kb for the three PARP1-null clones) had in general shorter telomeres than WT cells (median telomere length of 5.0 kb; Figure 4C). We did note that this phenotype was variable, *i.e.* some null clones were shorter than others, and most of the complemented clones did not restore the telomere length to WT levels. We attributed this to the inherent (and unfortunate) consequence of the clonal variation in telomere length that exists in human somatic cells. Thus, to properly analyze the capability of PARP1 expression to complement the *PARP1*^−/−^ cells, we created more independent complemented clones, along with empty vector clones, and re-screened their telomere lengths. While all (6/6) of the empty vector (EV)-containing clones had telomeres that were shorter than the *PARP1*-null parental cell line, only 5 of 17 of the PARP1-expressing clones were shorter. Correspondingly, 12 of the 17 *PARP1*-complemented clones remained at the parental (null) size or in a few cases actually showed telomere elongations, albeit with one exception, not to wild type length (Figure 4D). Thus, we concluded that PARP1 prevents abnormal telomere shortening, but it does not significantly contribute to telomere lengthening.

**Figure 5:**
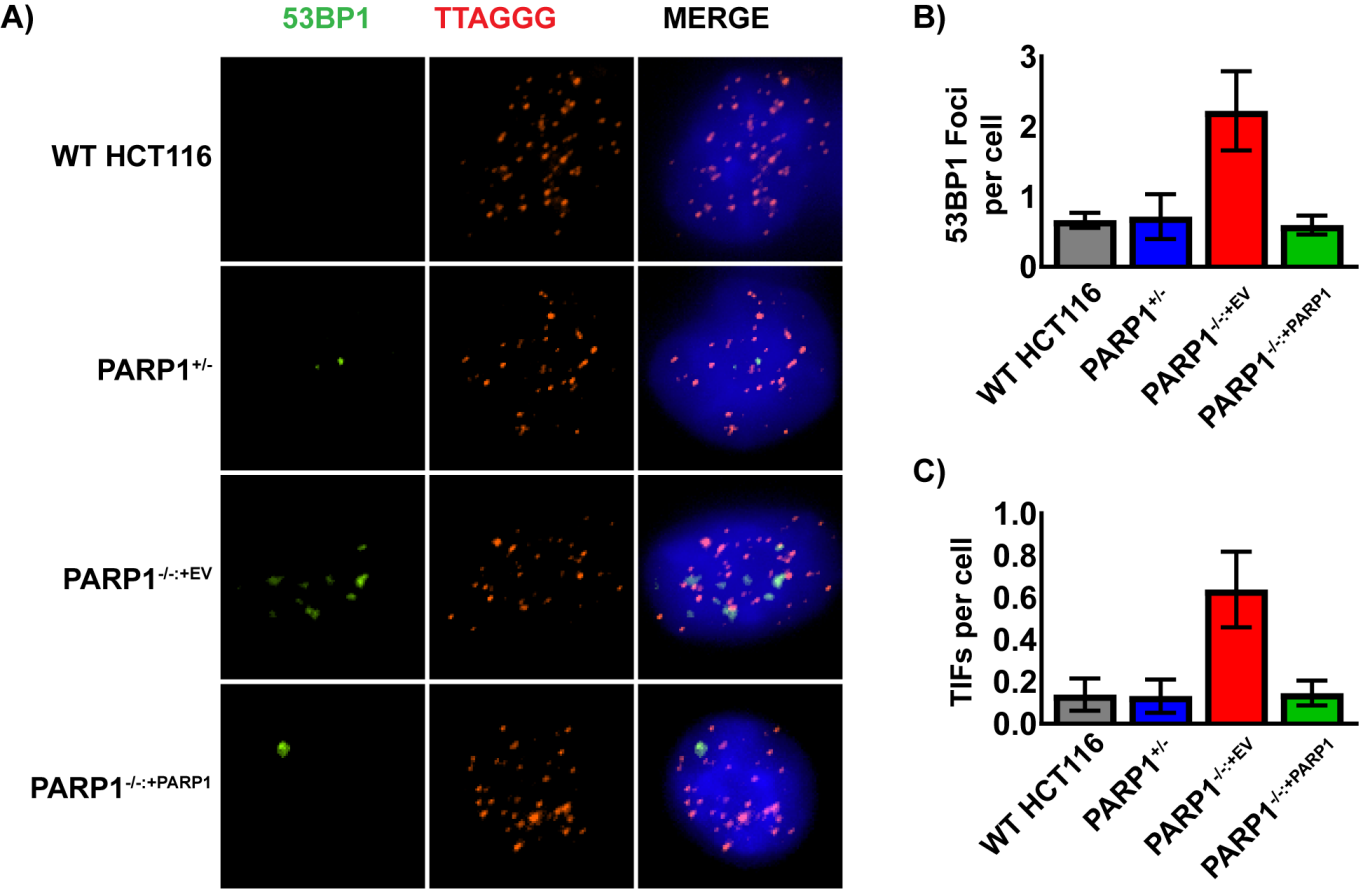
Spontaneous DNA damage foci in *PARP1*^−/−^ cells co-localize with telomeres **(A)** the indicated cells were immunostained for 53BP1 (green), fixed, and then subsequently probed for telomeric DNA with a PNA FISH probe (red) and for total DNA with DAPI (blue). *PARP1*^−/−^ cells had an elevated level of 53BP1 foci, which tended to colocalize with telomeric DNA. **(B)** The number of telomeric ends and overlapping 53BP1 foci (TIFs) on a per cell basis from images similar to panel (A) were averaged and graphed +/−1 standard deviation. *PARP1*^−/−^ cells had a statistically significant increase in the frequency of TIFs compared to the control cell lines. **(C)** Quantification of just 53BP1 foci/cell on a per cell basis from images similar to panel (A) were averaged and graphed +/−1 standard deviation. *PARP1*^−/−^ cells had an increased level of endogenous 53BP1 foci, which was indicative of DNA damage.

Given the aforementioned observed G_2_/M cell cycle arrest, we probed for any connection between the spontaneous DNA damage and the shortened telomere phenotype. Specifically, we utilized an IF-FISH assay, which combines immunofluorescence of proteins with FISH to co-visualize proteins and DNA sequences. We performed this assay in the various cell lines for both telomeric DNA and 53BP1, a common marker of DNA DSBs [59, 60]. In *PARP1*-null cells there was a higher spontaneous frequency of 53BP1 foci (Figure 5A, 5C), and these foci significantly (p > 0.05) co-localized with telomeric DNA (telomere dysfunction-induced foci, TIFs; Figure 5A, 5B). Importantly both the elevated 53BP1 foci and the TIFs could be completely suppressed by the re-expression of PARP1 in the *PARP1*-null cells (Figure 5). Together, these data demonstrated that the telomeres of *PARP1*-null cells are short and that they are prone to incurring significant amounts of DNA damage.

### PARP1 affects cellular immortalization

**Figure 6:**
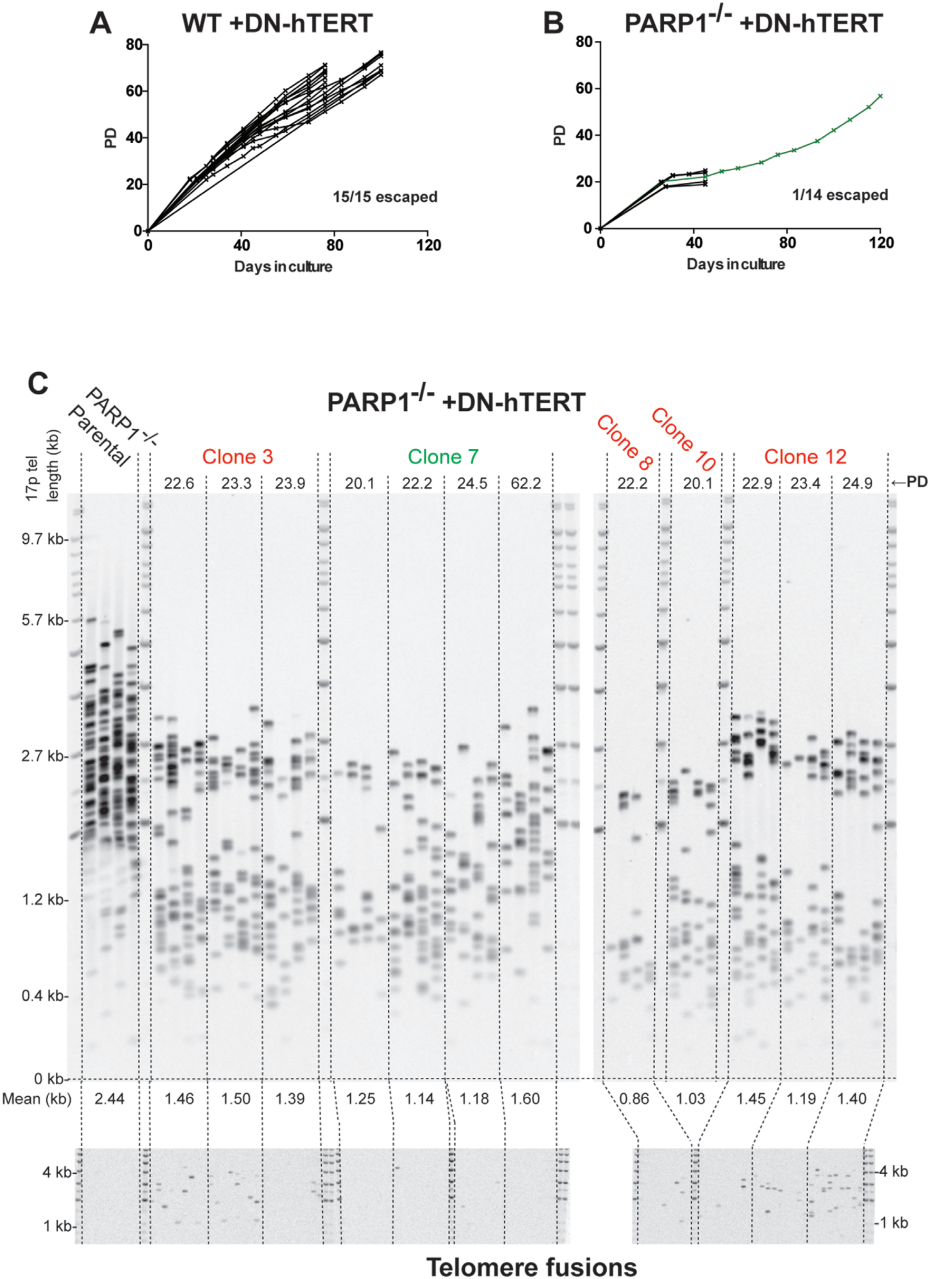
*PARP1*^−/−^ cells are severely compromised in surviving telomeric stress **(A)** growth curves plotting population doublings (PD) and days in culture. Each line represents an independent subclone. For some of the wild type clones the experiment was intentionally terminated after 80 days and for others only after 120 days. **(B)** growth curves plotting PD and days in culture for *PARP1*-null cells; only a single subclone (green line) survived beyond 40 days. **(C)** Top. STELA of the 17p telomere. The PD from the point of single cell cloning is shown above, while the mean (in kb) of the telomere length profiles is shown below. Bottom. Single-molecule telomere fusion analysis, using oligonucleotide PCR primers targeted to XpYp, 17p and the 21q family of related telomeres at the PD points as indicated.

To confirm and extend the conclusion that *PARP1*-null cells have dysfunctional telomeres, we next tested whether *PARP1*-null cells could survive a “telomere challenge”. In these experiments, a dominant negative telomerase (DN-h*TERT*) that suppresses endogenous telomerase activity was expressed in the cells. DN-hTERT expression results in telomere shortening and generally forces the cells into a “crisis” that is very much akin to the classic telomeric crisis that primary cells must overcome to enable cellular immortality [61]. Following the replicative erosion induced by the expression of the DN-hTERT, cells normally undergo a period of slow growth and genetic instability due to the resulting shortened telomeres, but ultimately re-establish wild-type telomerase expression and telomere maintenance [62, 63]. Indeed, when DN-h*TERT* was expressed in wild type cells, all of clones (15/15) analyzed escaped the subsequent crisis and continued to proliferate for at least 80 days, (or in some cases 100 days), after which point the experiment was intentionally terminated (Figure 6A). In stark contrast, only 1 of 14 *PARP1*-null clones was able to escape and immortalize (Figure 6B). To expand upon this observation, the *PARP1-null* clones were also subjected to a single telomere length analysis (STELA) after various population doublings (PDs) following the expression of DN-h*TERT*. STELA is a PCR-based technology using sub-telomeric anchored primers and linker primers ligated onto the ends of telomeres to analyze, at the single molecule level, the length of individual telomeres [62, 64]. Consistent with the TRF analysis (Figure 4C), the STELA analysis confirmed that the average telomere length of the *PARP1*-null parental population was a relatively short 2.44 kb (Figure 6C). DN-hTERT expression reduced this length further with many of the clones (#3, #7, #8, #10 and #12 are shown) having mean lengths between 0.86 to 1.50 kb after only ~20 PDs. Interestingly, the single *PARP1*-null clone that survived (clone #7) also showed early on (PDs 20.1 to 24.5) a significant reduction in telomere length indistinguishable from the clones that died although after PD 62.2 its telomeres began to subtly elongate. Telomere-specific single molecule PCR can also be used to detect telomere fusion events [62, 64]. No fusions were detected in the PARP1-null cell line in the absence of DN-hTERT expression (Figure 6C), which suggested that the increased SFEs (Figure 4B) and TIFs (Figure 5B) observed in this population was apparently not sufficient to induce telomeric fusion events. In striking contrast, telomeric fusions were detected with all of the *PARP1*-null clones expressing DN-hTERT although fewer translocations were detected in clone #7, which was the clone that ultimately survived (Figure 6C). Thus, in agreement with our end-joining data (Figure 3) *PARP1*-null cells apparently had no difficulty ligating their uncapped telomeres together. In summary, these data demonstrated that the telomere dysfunction observed in *PARP1*-null cells while insufficient to induce telomere fusions spontaneously, severely compromised the ability of the cells to re-establish telomere maintenance in the face of gradual telomere erosion.

## DISCUSSION

*PARP1* has been the subject of intense study in multiple model organisms. In spite of this, the molecular mechanism of PARP1 action in certain cellular transactions is still unclear. For example, while *PARP1* loss-of-function mutations were initially discovered to be synthetically lethal with *BRCA1* and *BRCA2* mutations [14], some cancer patients with such mutations have not benefited from PARP1 inhibition [65], and many tumors that are *BRCA1-* and *BRCA2*-proficient can likewise be sensitized by PARP1 inhibition [66, 67]. Thus, while there is no dispute that PARP1 inhibition can cause synthetic lethality in *BRCA1* and *BRCA2* mutant tumors, the molecular mechanism of that lethality is yet to be fully elucidated. One explanation for this ambiguity is that a common feature of PARP1 inhibitors is that they can have dominant-negative effects in cell lines that contain the target protein. While the results drawn from these experiments are not in any way invalid, it can be difficult to discern the effect of non-functional protein versus the absence of that protein. Here, we genetically ablated *PARP1* in a *BRCA1*- and *BRCA2*-positive cancer cell line, HCT116, to better understand the role of *PARP1* in an otherwise normal, albeit oncogenic, generic background.

HCT116 cells were selected as the cellular model for these experiments because they are diploid, have a stable karyotype and are wild-type for most DNA repair, DNA checkpoint and chromosome stability genes (reviewed in [68]). With that said, it should be noted that HCT116 cells are also mismatch repair defective and because of this deficiency the cells (especially if cultured for a long period of time — something that we explicitly avoid) have the tendency to accumulate genetic mutations [69]. In spite of this caveat, HCT116 cells have been utilized more than any other human cell line for carrying out reverse genetic gene editing experiments and have been proved to provide data that is comparable to many other human cell lines, including several that are non-tumorigenic [68]. Thus, while the findings presented here are potentially only relevant to this singular cancer cell line, we are confident the results accurately reflect the role of PARP1 in human cells.

Importantly then, in the *PARP1*-null cells we found little evidence of significant DSB repair defects, but rather observed an increase in cells accumulating in the G_2_/M checkpoint, likely as a result of endogenous DNA damage/short telomeres. We further showed that the DSBs that do appear tend to occur in telomeric DNA, which while likewise contributing to checkpoint activation, further limit the cell's proliferation and subsequent ability to handle telomere stress.

One novel finding from these studies is the demonstration that *PARP1* is a non-essential gene in human somatic cells. Our ability to isolate three independent *PARP1*-null clones is unequivocal evidence that PARP1 is not required for survival. With that said, there has never been, to our knowledge, a human patient described anywhere in the world who is/was *PARP1*-null. Indeed, our own gene targeting studies argue strongly that PARP-1, while not technically essential, is nonetheless so important that the development of a viable human is unlikely. Gene targeting is a completely egalitarian process and either allele in a diploid cell is as equally likely to be modified as the other [68]. During the second round of *PARP1* targeting however, 69 of 72 clones were re-targeted and only 3 of 72 correctly targeted clones resulted in the loss of the second *PARP1* allele (Table 1). This exceptional disequilibrium in the gene targeting frequency is a hallmark of genes that provide a significant growth disadvantage when absent [50–52]. The fact that any viable clones were obtained could be attributed to the possibility that the PARP proteins are functionally semi-redundant (discussed further below), and in rare cases other PARPs, such as PARP2 and PARP3 [70], could minimally fulfil the essential role of PARP1 in cells, although we note that there was no (detectable) PARylation activity in the PARP1-null cells (Figure 1D). Our subsequent demonstration that there are significant deficits in *PARP1*-null cells with telomere maintenance are completely consistent with this conclusion. Thus, the three independent *PARP1*-null clones notwithstanding, we predict that in the context of organismal development that *PARP1* will be essential and that *PARP1*-null patients will not be identified.

An additional key finding we present is the lack of a significant effect on A-NHEJ caused by the absence of *PARP1* (Figure 3; Supplementary Figure 3). While it is compelling that PARP1 inhibition can result in diminished end-joining activity in many cell types, we suggest that *PARP1* is not a critical A-NHEJ gene. Such mischaracterization has historical precedent, as *PARP1* has been previously mislabeled as a core BER gene [8]. This was originally suggested by the finding that *PARP1*^−/−^ murine embryonic fibroblasts (MEFs) were hypersensitive to BER-sensitizing alkylating agents, such as methyl-methane sulfonate [71]. However, subsequent investigation demonstrated that the inhibition of PARP1 was simply either trapping a BER-intermediate [12] or modulating the terminal ligation step [72] and that PARP1 was not an integral component of the BER machinery. Another potential contributor to the confusion regarding the role of PARP1 in A-NHEJ (and other cellular processes) is that PARP-inhibitors are often presumed to only inhibit PARP1, when the opposite is truer. Thus, since virtually all PARP inhibitors utilize NAD^+^ analogs to competitively bind and inhibit PARP enzymes, it is unsurprising that common inhibitors such as olaparib bind and inhibit most PARP family members, including PARP2 and PARP3, with equal affinity [73]. This is especially pertinent given the evidence that the PARPs have (at least partial) redundancies in their activities. For example, while *PARP1*-null mice are viable, *PARP1^−/−^:PARP2^−/−^* mice are not viable [74]. Moreover, singly mutant *PARP2*^−/−^ mice exhibit a modest radiation sensitivity, indicative of defects in DNA repair, which are presumed to overlap with PARP1 [74]. Similarly, PARP3 has been implicated in DNA repair and telomere integrity [75]. These functional redundancies have been biochemically confirmed (see for example, [76]) including most recently by mutating the NAD^+^ binding domains of PARP1, PARP2, and PARP3 and utilizing correspondingly distinct NAD^+^ analogs, which allowed the targets (some of which were overlapping) of each PARP to be identified [77]. Such partial functional redundancies underscore the necessity for careful genetic studies to affirm the role of each PARP in various cellular processes, in lieu of making general claims with nonspecific inhibitors.

Here, we hypothesize that the purported role of PARP1 in A-NHEJ may in fact be due to a combination of its SSB repair activity and perhaps non-redundant activities of other PARPs susceptible to inhibition. The lack of a strong A-NHEJ phenotype for *PARP1*^−/−^ human cells is consistent with emerging molecular evidence detailing the mechanism of A-NHEJ itself. Rather than a discrete sub-pathway of NHEJ, it now appears that A-NHEJ may be a HDR sub-pathway which is engaged when canonical HDR fails to find the appropriate homologous template for proper repair [78]. Several independent reports support this emerging theory. First, the requirement for microhomology at the ligation junction conceptually underlies a requirement for some degree of DNA-resection, followed by homology searching, both of which are much more akin to HDR than NHEJ. Moreover, reports have confirmed that the homology searching in A-NHEJ is dependent on the Meiotic Recombination 11/Radiation Sensitive 50/Nijmegen breakage syndrome 1 (*MRE11*/*RAD50*/*NBS1; MRN)* complex and C-terminal interacting protein (*CtIP*) [79, 80], as well as *BRCA1* [81], all of which are canonical HDR genes. In addition, the kinetics of A-NHEJ are similar to HDR and distinct from C-NHEJ [82]. *In toto*, these reports are consistent with A-NHEJ being a sub-pathway of HDR. If this model is true, then the key regulatory A-NHEJ genes are more than likely to be the upstream HDR repair genes, rather than *PARP1*. Thus, we suggest that while PARP1 inhibition affects A-NHEJ activity in certain experimental models [83–85], it is not a canonical A-NHEJ gene. With this said, a small, albeit significant and reproducible, deficit in A-NHEJ activity was observed in *PARP1*-null cells — intriguingly however, only when they were proficient for DNA-PK_cs_ (and therefore presumably proficient for C-NHEJ) (Figure 3, Supplementary Figure 3). This deficit is more compatible with the more widely accepted models of A-NHEJ being a salvage pathway for ineffective C-NHEJ [27]. Needless to say, these models are not mutually exclusive and A-NHEJ could be the salvage pathway for both unsuccessful HDR and C-NHEJ. In this scenario, the presence (or absence) of *PARP1* seems to impact the C-NHEJ salvage subpathway more than the HDR one. All of these models clearly deserve further experimentation/testing.

The role of *PARP1* in telomere maintenance has remained an ambiguous, yet intriguing, concept. To date, the majority of work has described a role for PARP1 in mediating aberrant DNA repair at uncapped or damage telomeres, specifically causing the fusion of sister-chromatid telomeres [41, 81, 86, 87]. Thus, these reports have implied that PARP1 is actively repressed from binding to functional telomeric DNA. Yet, other reports have indicated a functional interaction with Telomere Recognition Factor 2 (TRF2), a principal component of the Shelterin complex [43]. PARP1 was also independently identified as a Shelterin binding protein by an unbiased mass-spectroscopy approach [44]. Consistent with those reports is the fact that PARP1 possess a canonical TRF2-interacting motif (F/Y-X-L-X-P): _737_YTLIP_741_ [88]. Reports of the role of *PARP1* in MEFs are conflicting: certain *PARP1*^−/−^ MEFs exhibit telomere shortening [42, 43], while in other studies there was no appreciable telomere phenotype [46]. Our data strongly suggest that one of the critical roles of *PARP1* in human somatic cells is to maintain telomeric integrity. The most likely scenario is that PARP1 is preferentially recruited to telomeres, through its interaction with Shelterin, to help regulate the repair of SSBs caused, or encountered by, the DNA replication machinery [16]. Thus, the absence of PARP1 could result in the conversion of these telomeric SSBs to DSBs by DNA replication, resulting in a telomere shortening phenotype, DNA DSB signaling, and genomic instability — all of which we observed (Figures 4 and 5). Importantly, we do not suggest that PARP1 is a telomere lengthening protein; it does not function akin to telomerase and the re-introduction of PARP1 to *PARP1*-null cells did not result in extensive telomere elongation. Rather, we posit that the presence of PARP1 allows for longer telomeres to maintain their stability. This is evidenced by the variation we observed in the extent of the telomere length restoration in *PARP1*-null complemented cells. The absence of PARP1 does cause telomere shortening (albeit indirectly), but the re-expression of PARP1 in these cells only allowed cells to stabilize the longer telomeres that were subsequently generated by the clonal variation in telomerase-positive cancer cells (Figure 4). Thus, we conclude that PARP1's primary role is in preserving telomere length maintenance.

Recently, our laboratories have examined the contributions of the C-NHEJ and A-NHEJ pathways in facilitating the fusion of short dysfunctional telomeres in human cells following replicative erosion [62, 63]. These studies identified DNA Ligase 3 (*LIG3*) — a gene universally regarded as being A-NHEJ-specific — as being essential for cells to escape the subsequent crisis and survive [62]. Here we utilized this assay to determine if *PARP1*-null cells also have a role in telomere length maintenance, hypothesizing that if PARP1 was required for A-NHEJ, it would phenocopy the requirement for LIG3 in immortalization. Very surprisingly, we were able to demonstrate that although PARP1 had little impact on A-NHEJ (Figure 3) it nonetheless nearly completely phenocopied the requirement for LIG3 in immortalization (Figure 6). These results demonstrated that *PARP1* and *LIG3* do indeed share a strong genetic interaction for cellular immortalization caused by telomere shortening. At the same time, however, these results also contradicted our hypothesis and demonstrated that that genetic interaction is unlikely related to A-NHEJ. This conclusion suggests that SSB repair (a pathway that PARP1 and LIG3 also co-participate in) may be the culprit. We suggest that ssDNA lesions may accumulate in telomeric sequences (perhaps during the replication of the telomere) and the inability to accurately repair these lesions may facilitate chromosomal fusions. Although further experimentation will clearly be needed to clarify this issue the near inability of *PARP1*-null cells to survive crisis (Figure 6) is completely consistent with our posited role for PARP1 in maintaining telomeric homeostasis.

Finally, we note that our data have clinical implications. Thus, PARP1 inhibitors are currently being extensively utilized in the clinic. Our demonstration here that the absence of PARP1 in human cells leads to aberrant telomere maintenance suggests that there may be significant long-term repercussions to the chemical inhibition of PARP1 in human cells that might not be immediately evident.

## MATERIALS AND METHODS

### Cell culture

HCT116 cells were purchased from the ATCC and maintained in McCoy's 5A media supplemented with 10% FBS, 1% glutamine, and 1% penicillin/streptomycin. Cells were maintained in 10 cm plates and passaged every 3 to 5 days. To initiate telomere erosion in HCT116 cells expressing DN-h*TERT*, the cells were transduced with amphotropic retroviral vectors containing a DN-h*TERT* cDNA [89] as described [90]. For cell synchronization studies, cells were cultured for 16 hr in McCoy's 5A media containing 0.1% FBS, and subsequently grown in 2 mM thymidine for 24 hr. Cells were then released into complete McCoy's 5A media and collected by trypsinization at the indicated times.

### Gene targeting and *PARP1* knockouts

The *PARP1* gene knockout by exon replacement with rAAV was performed by rAAV-mediated gene targeting. Briefly, homology arms were constructed by PCR (Supplementary Table 1), flanked by a LoxP-IRES-Neo-LoxP cassette, and ligated into an rAAV production vector. Producer 293-AAV cells were co-transfected with pAAV Helper and pAAV Rep/Cap, as described [91]. Target wild type HCT116 cells (1 × 10^5^) were plated approximately 24 hr prior to rAAV-infection in a 6-well plate. Cells were infected with virus-containing media, and 48 hr-post infection, the cells were single-cell subcloned in the presence of 0.5 mg/mL G418. Drug-resistant colonies were collected ~2 weeks after infection, and the correct replacement of exon 4 was screened by PCR (Supplementary Table 1). Correctly targeted clones were plated (1 × 10^5^) and infected with an adenoviral vector expressing the Cre-recombinase to remove the drug selectable marker by Cre-recombination. Cells were again single cell sub-cloned, and screened for correct Cre recombination events by PCR flanking exon 4. This process was repeated stepwise to inactivate the second PARP1 allele.

### DNA repair assays

All transfections were performed on 5 × 10^5^ cells with Lipofectamine 3000 in 6-well plates, which had been subcultured 24 hr prior to transfection. For the A-NHEJ reporter assay, we transfected 2.5 μg of linearized pDVG94 into target cells and allowed 24 hr for repair. The cells were subsequently collected by trypsinization, and re-circularized plasmids were recovered using conventional small-scale plasmid DNA isolation, as proper repair of the linearized junction by human cells creates a circularized DNA product which is accordingly recoverable. Repaired DNA junctions were PCR amplified using the FM30 and DAR5 primers [54]. PCR products were then digested with the *BstXI* restriction enzyme. Digested PCR products were resolved by electrophoresis on a 6% polyacrylamide gel. The gel was then stained with SybrGold and imaged on a Typhoon FLA 9500 imager.

For the FACS-based NHEJ reporter assay, we first subcloned the *ISce-I* coding sequences from an expression plasmid [92] and added a C-terminal T2A-mCherry epitope by fusion PCR. We then cloned this expression construct into a pcDNA 3.1 expression vector. For each NHEJ FACS assay, 1.25 μg of pGEM-Ad2-EGFP was co-transfected with the *ISce-I*-T2A-mChery plasmid into 5 × 10^5^ cells in 6-well plates. 24 hr following transfection, cells were collected by trypsinization, fixed with 4% formaldehyde, and subjected to FACS analysis.

### Telomere/terminal restriction fragment (TRF) assay

Genomic DNA was extracted from ~1 × 10^7^ cells, and 50 μg of genomic DNA was digested with *HinfI* and *RsaI*, as described [93]. For each sample, 12 μg of digested genomic DNA was resolved overnight on a 0.7% agarose 1 x TBE gel. This gel was depurinated, denatured, and neutralized, followed by overnight capillary transfer to a nitrocellulose membrane. The membranes were pre-hybridized for 1 hr with Church's buffer, then hybridized with a γ-P^32^-endlabeled telomere probe in 4X SSC at 55°C overnight. Membranes were washed 3 times with 4X SSC and once with 4X SSC + 0.1% SDS, each for 30 min, exposed to a phosphorimaging screen, and detected and quantitated with a Typhoon phosphoimager.

### Immunofluorescence and telomere FISH (IF-FISH)

This assay was performed as described [94]. Briefly, cells (1 × 10^5^) were plated on chamber slides, and allowed to grow for 24 hr. Cells were washed once with PBS, then fixed with 4% formaldehyde in 1X PBS. Blocking and RNaseA treatment (0.1 mg/mL) were performed in antibody dilution media (ABDIL; 20 mM Tris-HCl pH 7.4, 0.2% fish gelatin, 2% BSA, 0.1% Triton X-100, 150 mM NaCl, and 0.1% sodium azide) at room temperature for 30 min. Cells were stained with a 53BP1 antibody (Ab36823; Supplementary Table 2), which was diluted in ABDIL for 1 hr, washed 3 times with 1X PBS + 0.1%Tween-20 (PBST), and incubated with an Alexa-488 goat IgG secondary antibody diluted in ABDIL for 1 hr. Cells were washed in PBST, fixed with 4% formaldehyde and prepared for FISH hybridization. A Telo-C PNA probe was hybridized to the slides at 80° in hybridization buffer (10 mM Tris-HCl pH 7.4, 4 mM Na_2_HPO_4_, 0.5 mM citric acid, 1.25 mM MgCl_2_, 0.25% blocking reagent and 70% formamide). Slides were washed, counterstained with DAPI, and mounted with ProLong Gold (ThermoFisher). Microscopy was performed with a Nikon-TiE deconvolution bright-field microscope with a 60X objective.

### STELA and telomere fusion assay

Telomere length was determined using 17p STELA as described [94]. Briefly, DNA was extracted using proteinase K, RNase A, phenol/chloroform protocols and quantified by Hoechst 33258 fluorometry (Bio-Rad) before dilution to 10 ng/μL in 10 mM Tris-HCl, pH 7.5. A total of 10 ng of DNA was further diluted to 250 pg/μL in a volume of 40 μL containing 1 μM Telorette2 linker and 1 mM Tris-HCl, pH 7.5. Multiple polymerase chain reactions (PCRs; 6 reactions per sample) were carried out for each test DNA in 10-μL volumes with 250 pg of DNA, 0.5 μM of the telomere-adjacent and Teltail primers, 75 mM Tris-HCl, pH 8.8, 20 mM (NH4)_2_SO_4_, 0.01% Tween-20, 1.5 mM MgCl_2_, and 0.5 U of a 10:1 mixture of Taq (ABGene) and Pwo polymerase (Roche Molecular Biochemicals). The reactions were cycled with an MJ PTC-225 thermocycler (MJ Research). The DNA fragments were resolved by 0.5% Tris acetate ethylenediaminetetraacetic acid agarose gel electrophoresis, and detected by Southern blot hybridization with random-primed α-^33^P–radiolabelled (GE Healthcare) TTAGGG repeat probe together with probes to detect the 1-kb (Stratagene) and 2.5-kb (Bio-Rad) molecular weight markers. The hybridized fragments were detected by phosphorimaging with a Molecular Dynamics Storm 860 phosphorimager (GE Healthcare). The molecular weights of the DNA fragments were calculated using the Phoretix 1D quantifier (Nonlinear Dynamics).

The telomere fusion assay was carried out as described [95]. PCR reactions were carried out each containing 100 ng of DNA with XpYpM, 17p6 and 21q1 PCR primers. Fusion molecules were detected by Southern blotting and hybridization with the XpYp, 17p and 21q telomere-adjacent probes as described [95].

## SUPPLEMENTARY MATERIALS FIGURES AND TABLES


