# Supplementary material for: PARP1 is required for preserving telomeric integrity but is dispensable for A-NHEJ
Source: Oncotarget. 2018 Oct 5;9(78):34821–37. doi: 10.18632/oncotarget.26201 (PMC6205175; doi:10.18632/oncotarget.26201)
Supplement: Supplementary file 1 [file oncotarget-09-34821-s001.pdf]

PARP1 is required for preserving telomeric integrity but is dispensable for A-NHEJ

SUPPLEMENTARY MATERIALS

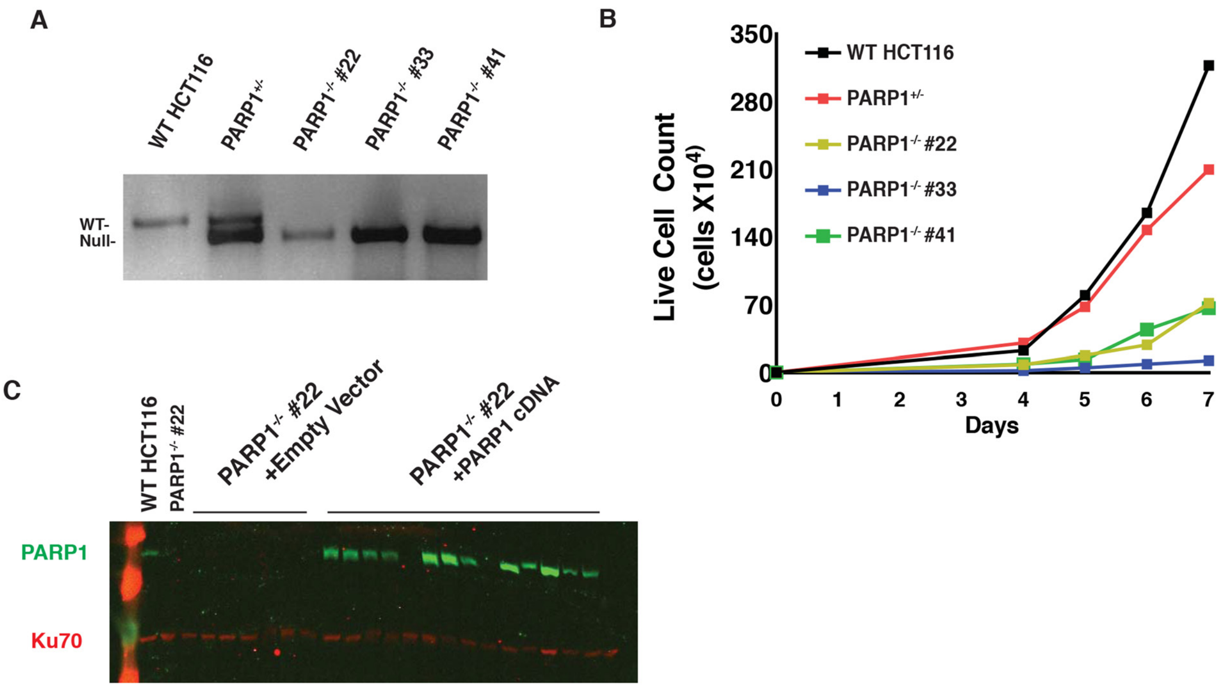

Supplementary Figure 1: Characterization of the 3 independent *PARP1*-null cell lines. **(A)** a PCR analysis is shown using primers that flanked exon 4 (see Figure 1A). The wild type (WT) allele PCR product migrates slightly slower than the PCR product corresponding to the null allele. The genotypes and clone designations are shown above the gel. **(B)** *PARP1*-null cell lines show a significant proliferation defect. Cells from the indicated cell lines were seeded into dishes and then the total cell number was monitored by trypan blue staining at days 4 to 7 post plating. **(C)** a Western blot analysis of the *PARP1*-null subclone #22 cell line. PARP1 protein is shown in green and as a loading control, the Ku70 protein is shown in red. The red smears on the far left in the unmarked lane are protein standard markers. *PARP1*-null clones expressing only the empty vector contained no detectable PARP1 protein whereas the complemented clones expressed various levels of PARP1 protein.

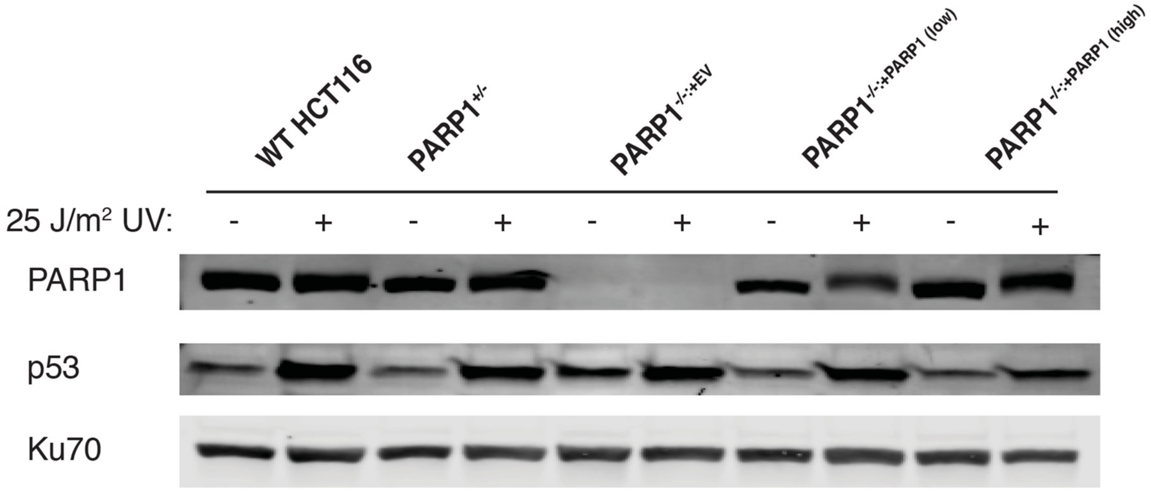

Supplementary Figure 2: Levels of p53 protein in the *PARP1*-null cells. The indicated cell lines were exposed to +/- 25 J/m^2 UV radiation, and whole cell lysates were collected 24 hr later. Western blot analyses were subsequently performed for PARP1, p53, and, as a loading control, Ku70. The *PARP1*$^{-/-}$ cells exhibited elevated levels of spontaneous p53. However, all the cell lines were capable of additional increased p53 expression following UV irradiation.

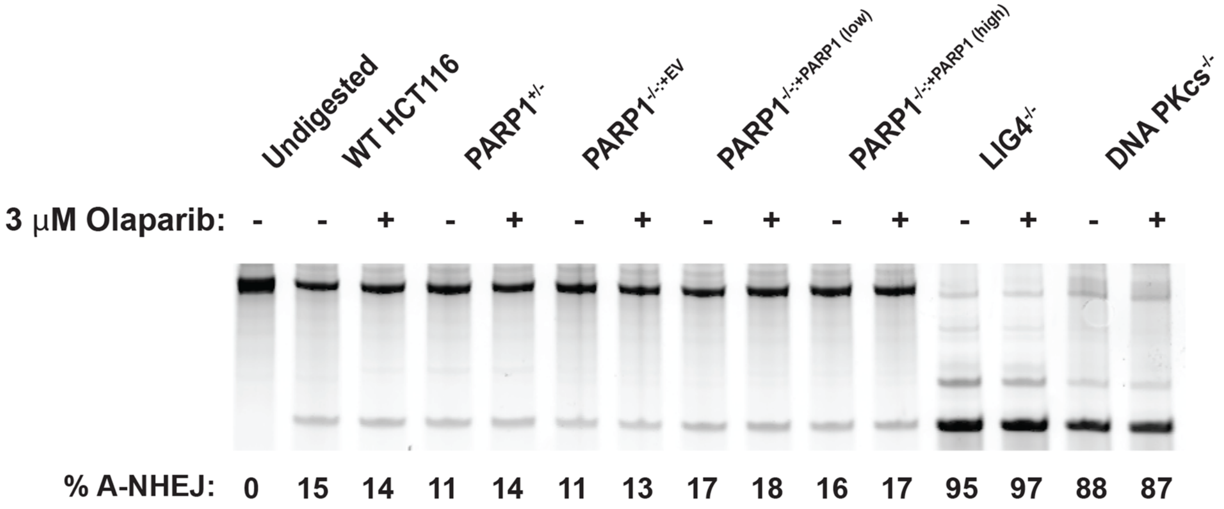

Supplementary Figure 3: PARP1 inhibition does not affect A-NHEJ activity. The indicated cell lines were either left untreated (-) or treated (+) with 3 μM of olaparib, a PARP1 inhibitor, for 4 hr, and then transfected with a linearized pDVG94 plasmid. The indicated cell lines were allowed 24 hr to repair the linearized template (still in the presence or absence of inhibitor), and then plasmids were extracted, and the region spanning the cut-site was amplified by PCR, digested with the *BstXI* restriction enzyme and then analyzed by gel electrophoresis. The % of A-NHEJ, as described in the legend to Figure 3B, was determined and is shown below the gel. *N.B.* The sensitivity of HDR-defective (*FANCA*-null) cells to the same concentration of olaparib was used as a positive control to demonstrate that the olaparib used in this experiment was active (data not shown).

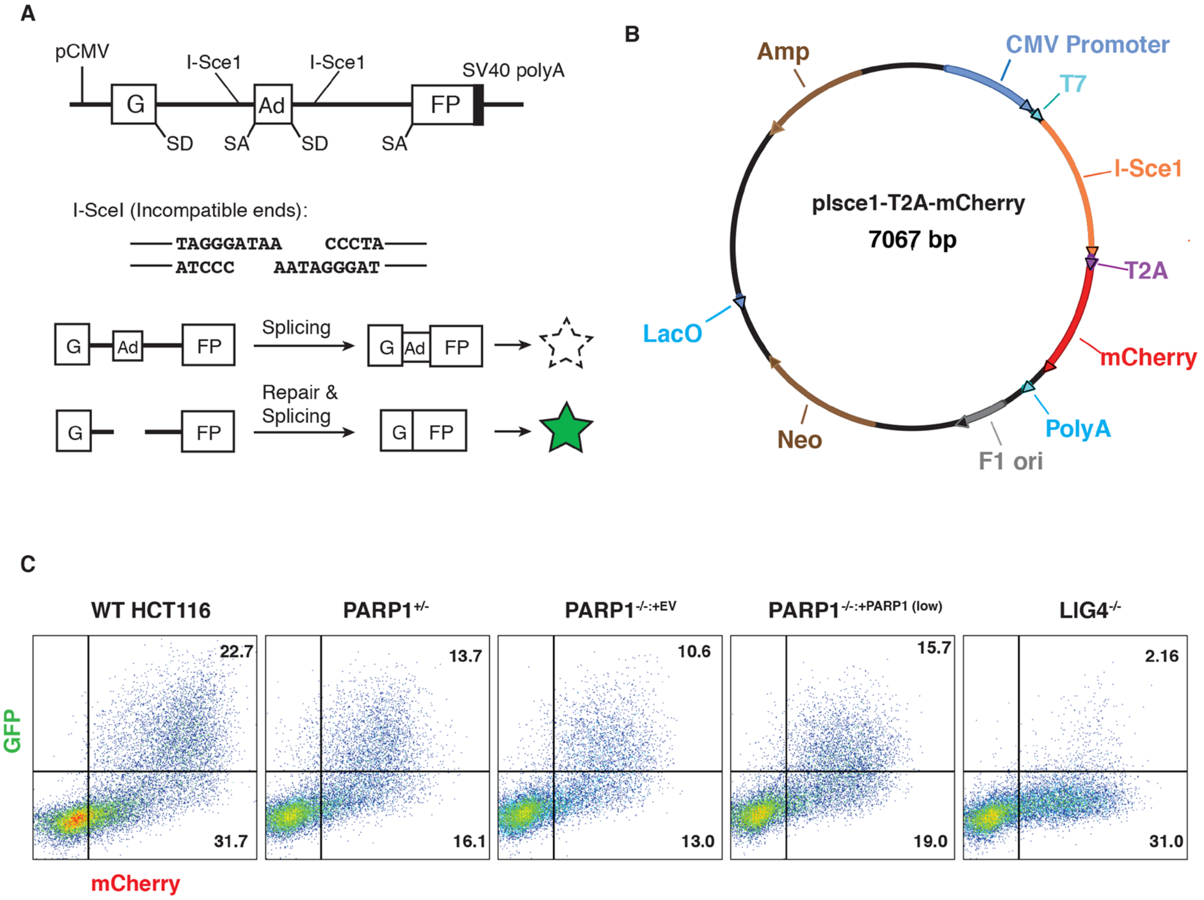

$$\% \text{ Repair} = \frac{\#\ \text{GFP cells}}{\#\ \text{GFP} + \text{mCherry cells}}$$

Supplementary Figure 4: The absence of *PARP1* does not affect C-NHEJ activity. (A) schematic of the pGEM-Ad2-EGFP NHEJ reporter. The rectangular boxes correspond to the indicated protein open reading frames. SD and SA indicate splice donor and acceptor sequences. This reporter must be linearized and repaired within the indicated intronic regions (resulting in the lack of retention of the Ad sequences), to enable GFP expression. **(B)** a schematic of the mCherry vector used in this assay to control for transfection efficiency. **(C)** a representative set of FACS images for the indicated cell lines. The numbers within the panels represent the fraction of cells that were GFP or RFP (mCherry) positive, respectively. The formula for determining the percent repair is shown below the panels. Only the *LIG4*-null cells were deficient in this assay (see Figure 3D).

Supplementary Table 1: Table of PCR primers

Primer Name	Sequence 5’ > 3'
LarmF	atacatacgcggccgcCTAATGTGACATGCACATATTAATT
LarmR	ctatacgaagttatggatccactagtctagaGAAAAACCATATGTGGTACCAAGG
RarmF	cattatacgaagttatccaaattttgaattcGTTCAAGGGAGGCCCAAAGGCCCTAC
RarmR	atacatacgcggccgcCTGACAAGAAGTGACAGTCAAAG
Exon4SeqF	CCTTGATGACCTGAGAGAGC
Exon4SeqR	GGAATAGACCACCAGGAAGCAG

*lowercase letters: cloning sequence adapters, UPPERCASE-prime genomic DNA.

Supplementary Table 2: List of antibodies used in Western analyses

Protein Target	Supplier Catalog Number	Dilution
PARP1	Sc-8007	1:300
p53	SC-126	1:1000
KU70	Sc-9033	1:2000
H2AX	A300-081A	1:1000
